# Ubiquitination and SUMOylation in Telomere Maintenance and Dysfunction

**DOI:** 10.3389/fgene.2017.00067

**Published:** 2017-05-23

**Authors:** Zeliha Yalçin, Carolin Selenz, Jacqueline J. L. Jacobs

**Affiliations:** Department of Molecular Oncology, Netherlands Cancer InstituteAmsterdam, Netherlands

**Keywords:** ubiquitin, SUMO, telomere maintenance, telomere dysfunction, DNA damage, DNA repair, shelterin, telomerase

## Abstract

Telomeres are essential nucleoprotein structures at linear chromosomes that maintain genome integrity by protecting chromosome ends from being recognized and processed as damaged DNA. In addition, they limit the cell’s proliferative capacity, as progressive loss of telomeric DNA during successive rounds of cell division eventually causes a state of telomere dysfunction that prevents further cell division. When telomeres become critically short, the cell elicits a DNA damage response resulting in senescence, apoptosis or genomic instability, thereby impacting on aging and tumorigenesis. Over the past years substantial progress has been made in understanding the role of post-translational modifications in telomere-related processes, including telomere maintenance, replication and dysfunction. This review will focus on recent findings that establish an essential role for ubiquitination and SUMOylation at telomeres.

## Introduction

Genome stability is essential for cells to function properly and ensure the survival of an organism. At the ends of chromosomes this stability is maintained by telomeres. In vertebrates telomeres consist of long double-stranded stretches of TTAGGG repeats, ending in a ∼50–500 base pair overhang of the G-rich 3′-strand (Palm and de Lange, 2008). The protein complex shelterin, consisting of TRF1, TRF2, TIN2, POT1, TPP1 and RAP1, binds to telomeric repeats and mediates the formation of a telomeric loop (T-loop) in which the single-stranded 3′-overhang is concealed in a D-loop (Griffith et al., 1999; Doksani et al., 2013). This is necessary to prevent DNA damage response (DDR) and repair mechanisms from recognizing the single-stranded DNA (ssDNA) overhang. Due to incomplete replication of chromosome ends, each round of DNA replication progressively shortens linear chromosomes, risking loss of essential genes or important regulatory regions. To prevent this, telomeres act as a buffer region to maintain genome integrity (Harley et al., 1990). Replication of telomeres is initiated by the polymerase alpha-primase (PP) complex, which consists of subunits that have polymerase and primase activity (Pellegrini, 2012). During lagging-strand synthesis the ultimate RNA primer is removed, but cannot be replaced with DNA, resulting in an overhang. Additionally, leading-strand synthesis creates a transient blunt end that is processed by nucleases to generate a short 3′-overhang. Therefore, incomplete replication of the lagging strand and resection of the leading strand result in 3′-overhang generation, which contributes to telomere shortening and is known as the “end-replication problem” (Chow et al., 2012; Chen and Lingner, 2013; Martinez and Blasco, 2015). Besides the end-replication problem, replication at telomeres is extra challenging because of topological barriers, such as the T-loop and the presence of G-quadruplexes. Proper telomere replication requires G-quadruplex resolution and suppression of G-quadruplex formation by the helicases BLM, DNA2, WRN and RTEL1, and also T-loop disassembly by WRN and RTEL1 (Uringa et al., 2012; Vannier et al., 2012, 2013; Crabbe et al., 2004; Edwards et al., 2014; Martinez and Blasco, 2015).

In many stem cells and in the majority of cancer cells telomere shortening is, respectively, partially or completely, compensated by telomerase. Telomerase consists of a telomerase reverse transcriptase (TERT) catalytic subunit and an RNA template (TERC) that add *de novo* TTAGGG repeats to chromosome ends. Telomerase is recruited to telomeres via TIN2-TPP1, whereby TPP1 promotes telomerase activity and telomere extension. First, the 3′-strand is extended by TERT using TERC as the complementary template to synthesize telomeric repeats. Subsequently, in humans, the CST complex binds to this newly generated 3′-strand and recruits the PP-complex to sequentially fill-in the 5′-strand (Greider and Blackburn, 1985; Reveal et al., 1997; Huang et al., 2012; Nandakumar and Cech, 2013). Alternatively, cancer cells that do not express telomerase can counteract telomere shortening by activating the alternative lengthening of telomeres (ALT) pathway. This pathway makes use of homologous recombination (HR)-dependent exchange/synthesis of telomeric DNA. Telomeric DNA can, for example, be copied from a nearby template (the same telomere or the sister telomere), but also from a more distant template such as a telomere from another chromosome (Pickett and Reddel, 2015). In addition, specialized types of promyelocytic leukemia (PML) bodies, so-called ALT-associated PML bodies (APBs), are essential for telomere maintenance in ALT-positive cells (Yeager et al., 1999). Telomeres cluster in APBs, which in addition to telomere-binding factors and telomeric DNA also contain proteins involved in HR to perform ALT (Pickett and Reddel, 2015). HR is a DNA repair pathway that outside of telomeres is used to correctly repair a DNA break by using the sister chromatid as template.

However, when cells proliferate in the absence of telomerase or ALT, telomeres become critically short and shelterin is not able to bind to chromosome ends in sufficient amounts (Nandakumar and Cech, 2013). This leads to initiation of DDR signaling and DNA repair activities that can impair cell proliferation and harm genome stability (d’Adda di Fagagna et al., 2003; Jacobs and de Lange, 2005; Davoli and de Lange, 2011; Jacobs, 2013). Also, when replication at telomeres stalls because of topological barriers that cannot be resolved by helicases, a DDR is activated to restart replication through HR (Badie et al., 2010; Tacconi and Tarsounas, 2015; Zimmer et al., 2016). The DDR and DNA repair mechanisms at dysfunctional telomeres are tightly regulated by post-translational modifications (PTMs). In addition, telomere maintenance and protection, which function to prevent DDR initiation at telomeres, are also affected by PTMs, including ubiquitination and SUMOylation (Peuscher and Jacobs, 2012).

In the process of ubiquitination, the 76 amino acid protein ubiquitin is covalently conjugated via its C-terminus to the 𝜀-amino group of lysine residues or to the N-terminus of a target protein. Ubiquitination is implicated in many cellular pathways in almost all eukaryotic organisms and can target proteins for proteasomal degradation or affect their activity, localization and interaction with other molecules. The attachment of ubiquitin occurs via an enzymatic cascade consisting of E1 ubiquitin-activating, E2 ubiquitin-conjugating and E3 ubiquitin-ligating enzymes (Ciechanover et al., 1982; Hershko et al., 1983; Komander and Rape, 2012). Moreover, ubiquitin itself can also be ubiquitinated at its N-terminal M1 residue and at one of its seven internal lysine residues K6, K11, K27, K29, K33, K48 and K63. Therefore, ubiquitin-chains with many different linkages can be formed, significantly increasing their signaling potential and specificity. For example, K48-linked chains usually target proteins for proteasomal degradation (Komander and Rape, 2012). Ubiquitination is reversible through the action of deubiquitinating enzymes (DUBs), of which approximately 100 are known in humans. DUBs are able to cleave off an individual ubiquitin or break the bonds within the ubiquitin-chain, allowing for removal and editing at these sites (Komander et al., 2009).

Another PTM that is very similar to ubiquitination is SUMOylation. In this process, a small ubiquitin-related modifier (SUMO) protein is conjugated to target proteins. This also occurs via an enzymatic cascade, mediated by E1, E2 and E3 SUMO enzymes, which conjugate SUMO to the substrate protein in the same manner as ubiquitin (Johnson, 2004). Additionally, deSUMOylating enzymes can reverse this process (Mukhopadhyay and Dasso, 2007). In contrast to the ubiquitin system, for which over 600 E3 ligases are known to exist in humans, only a few SUMO ligases have been identified so far. In addition, multiple SUMO isoforms exist, with SUMO1 (101 amino acids), SUMO2 (95 amino acids) and SUMO3 (103 amino acids) being the ones that have been studied best (Cubenas-Potts and Matunis, 2013). In contrast to ubiquitin-chains, SUMO-chains do not directly target proteins for proteasomal degradation, but can prime the target for ubiquitin ligase-mediated degradation. Moreover, SUMOylation can influence protein activity, localization and interactions between proteins containing SUMO-interacting motifs (SIMs) (Geiss-Friedlander and Melchior, 2007; Kerscher, 2007). In the past years evidence increased for crucial roles of ubiquitination and SUMOylation in the cellular response to telomere dysfunction that potentially leads to genomic instability. Therefore, the aim of this review is to provide an overview of new findings obtained about ubiquitination and SUMOylation involved in telomere maintenance, replication and dysfunction.

## Telomere Maintenance: Shelterin in Control

**FIGURE 1:**
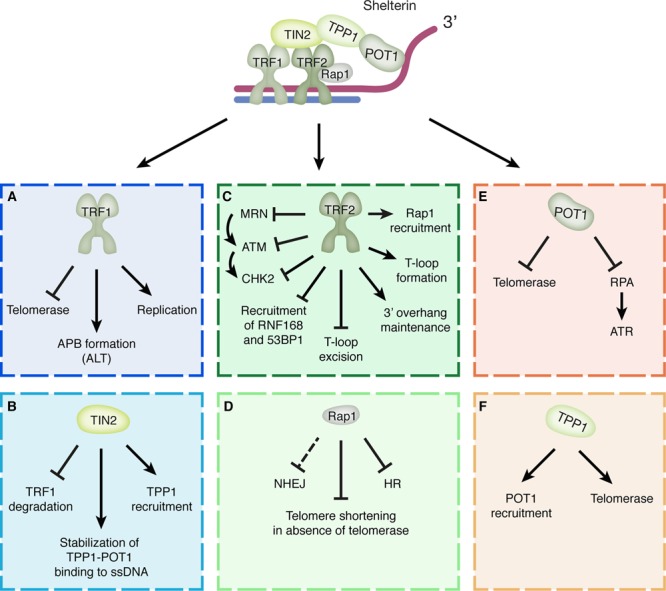
**Shelterin components and their functions in telomere protection. (A)** TRF1 facilitates telomere replication, restricts telomerase access and promotes the formation of APBs associated with ALT. **(B)** TIN2 recruits TPP1 to telomeres, stabilizes TPP1-POT1 binding to the ssDNA and prevents proteasomal degradation of TRF1. **(C)** TRF2 is involved in T-loop formation and stabilization, prevents T-loop excision, promotes maintenance of the 3′-overhang, recruits RAP1 to telomeres and prevents the recruitment of RNF168 and 53BP1. Furthermore, TRF2 interferes with ATM signaling by (1) preventing binding of the MRN complex and thereby activation of ATM, (2) binding ATM and interfering with its activation directly and (3) interacting with CHK2 and interfering with its phosphorylation. **(D)** RAP1 inhibits HR at telomeres and prevents telomere shortening in the absence of telomerase. In addition, RAP1 appears able to provide a back-up mechanism for inhibition of NHEJ when TRF2 function is impaired. **(E)** POT1 inhibits RPA binding and access of telomerase to the telomere single-stranded 3′-overhang. **(F)** TPP1 recruits POT1 to telomeres and stimulates the recruitment and activity of telomerase.

Aberrant telomere function can have severe cellular consequences by leading to genomic instability, cellular senescence and early apoptosis. Therefore, tightly regulated telomere maintenance is required to ensure protection of chromosome ends. The most significant complex involved in telomere maintenance and protection is shelterin (**Figure 1**). Shelterin governs telomere maintenance and protection in essentially three main ways: (1) by preventing activation of the DDR and DNA repair mechanisms at telomeres, (2) by facilitating telomere replication and (3) by regulating telomerase-mediated telomere elongation. The shelterin components TRF1 and TRF2 directly interact with telomeric DNA and are structurally very similar. Although, both proteins have a TRF homology (TRFH) domain and a SANT/Myb DNA-binding domain, TRF1 and TRF2 do not physically interact and have separate functions (Stewart et al., 2012; Doksani and de Lange, 2014). TRF1 has been shown to be required for proper telomere replication, for example by recruiting the necessary helicases, such as BLM, and for restricting telomerase access to the telomeres (Sfeir et al., 2009). In contrast, TRF2 is involved in T-loop formation and stabilization, prevents T-loop excision and promotes maintenance of the 3′-overhang by recruiting the Apollo nuclease. It is also essential for inhibition of the ATM kinase to repress DNA damage signaling and inhibit classical non-homologous end-joining (c-NHEJ), an error-prone repair pathway that promotes ligation of broken DNA ends (Karlseder et al., 2004; Wang et al., 2004; Denchi and de Lange, 2007; Wu et al., 2010; Doksani et al., 2013; Okamoto et al., 2013). TRF2 interacts with the shelterin component RAP1 and recruits it to the telomeres. Unlike for TRF2, the contribution of RAP1 to protection of mammalian telomeres against NHEJ is less evident and only noticeable in experimental conditions where RAP1 is artificially recruited to TRF2-depleted telomeres or TRF2 function is partially compromised (Sarthy et al., 2009; Kabir et al., 2014; Benarroch-Popivker et al., 2016). A more obvious role for RAP1 appears to be in protecting telomeres against HR. RAP1 deletion in a *Ku70*^-/-^ background resulted in increased telomere-sister chromatid exchanges, indicating that RAP1 represses HR (Sfeir et al., 2010). Furthermore, telomeres devoid of RAP1 and the N-terminal basic domain of TRF2 are rapidly resected by HR factors, resulting in telomere loss and telomere-free fusions (Rai et al., 2016). Finally, mice lacking both RAP1 and telomerase show increased telomere shortening and progressively decreased survival compared to single telomerase knockout mice (Martinez et al., 2016). Thus RAP1 seems to aid both in protecting telomeres from DNA repair activities and in maintaining telomeres in absence of telomerase. Much of the mechanistic basis for these roles of RAP1 still remains to be discovered.

TRF1 and TRF2 are bridged by TIN2, which acts as a linker protein. This ensures the integrity of the whole shelterin complex by connecting not only the double-strand DNA-binding TRF1 and TRF2, but also by linking TRF1 and TRF2 to the ssDNA-binding TPP1-POT1 heterodimer. In addition, TIN2 also recruits TPP1 to the telomere and stabilizes TPP1-POT1 binding to the ssDNA (O’Connor et al., 2006; Palm and de Lange, 2008). POT1 is the only shelterin component that directly interacts with the single-stranded 3′-overhang through its two OB-fold domains (Zhong et al., 2012). It is recruited to the telomeres by interacting with TPP1 (Loayza and De Lange, 2003; Liu et al., 2004). Binding of the POT1-TPP1 complex to telomeres contributes to telomere maintenance by preventing other factors, such as replication protein A (RPA), from binding to the 3′-overhang and promoting DNA damage signaling. In addition, both POT1 and TPP1 are involved in the regulation of telomerase activity, showing opposing effects on telomerase (Stewart et al., 2012). Whereas POT1 negatively regulates telomerase binding by making the 3′-overhang inaccessible, TPP1 has been observed to promote recruitment of telomerase and stimulate its activity (Stewart et al., 2012). Although it is not yet exactly known how these opposing functions of POT1 and TPP1 are coordinated, recent work suggests that POT1 inhibits telomerase recruitment by suppressing phosphorylation of TPP1 at Ser255 by the M-phase kinase NEK6 (Hirai et al., 2016). According to the proposed model, POT1 might dissociate from the telomeres during replication, after the T-loop is dismantled, thereby relieving the inhibitory effect of POT1 on TPP1. This could then allow for phosphorylation of TPP1 and thereby promote recruitment of telomerase to telomeres to maintain the telomeric sequence (Hirai et al., 2016).

### Regulation of TRF1 by Ubiquitin

**FIGURE 2:**
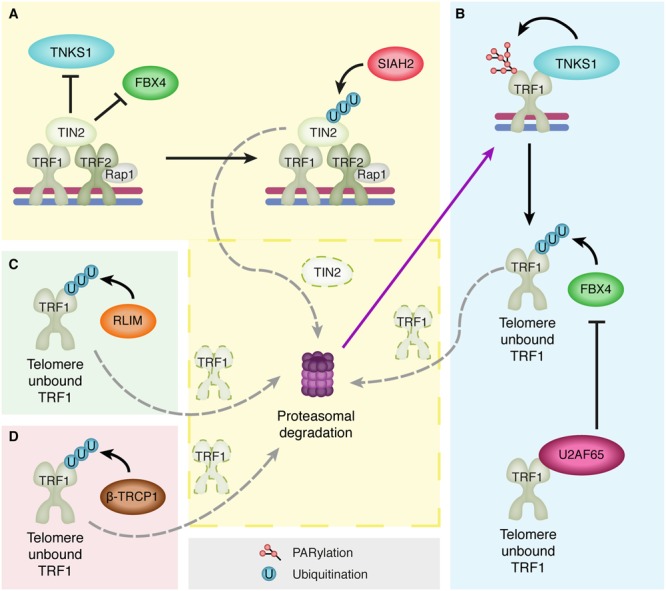
**The multilevel regulation of TRF1 degradation. (A)** TIN2 protects TRF1 from proteasomal degradation by preventing binding of Tankyrase 1 (TNKS1) and the E3 ligase FBX4 to TRF1. However, TIN2 itself is also targeted for proteasomal degradation by ubiquitination through the E3 ligase SIAH2, which releases the inhibition on TNKS1 and FBX4. **(B)** Subsequently, TNKS1 can PARylate TRF1 resulting in its dissociation from the telomeric DNA. This allows for FBX4-mediated TRF1 ubiquitination and proteasomal degradation. However, U2AF65 also binds telomere-unbound TRF1, which inhibits FBX4 binding and subsequent TRF1 degradation. **(C,D)** The E3 ligases RLIM **(C)** and β-TRCP1 **(D)** also ubiquitinate telomere-unbound TRF1 and promote its proteolysis.

In recent years, shelterin components have been shown to be regulated by PTMs. TRF1 levels are regulated by ubiquitin-mediated degradation that is facilitated by three E3 ligases: RLIM (RING H2 zinc finger or RNF12) and the F-box proteins FBX4 and β-TRCP1 (Lee et al., 2006; Her and Chung, 2009; Wang C. et al., 2013) (**Figure 2**). RLIM binds to a region between the dimerization and Myb domain of TRF1 and targets TRF1 for proteasomal degradation (Her and Chung, 2009). Similarly, FBX4 binds to the N-terminal region of the TRFH dimerization domain of free TRF1 (unbound to telomeric DNA) and also targets TRF1 for proteasomal degradation (Lee et al., 2006). When either RLIM or FBX4 is depleted TRF1 levels are stabilized, resulting in impaired cell growth and a decrease in telomere length, as TRF1 binding to telomeres is inhibitory toward telomerase. Accordingly, upon RLIM or FBX4 overexpression, levels of TRF1 decline, indicating a negative regulatory role for RLIM and FBX4 on TRF1 (Her and Chung, 2009). Moreover, a recent study has identified a novel TRF1-interacting protein that prevents FBX4 binding to TRF1. The splicing factor U2AF65 acts as a positive regulator of TRF1 by preventing FBX4-mediated ubiquitination and subsequent degradation of TRF1 (Kim and Chung, 2014). It has been proposed that U2AF65 only interacts with telomere unbound TRF1, as U2AF65 interacts with the Myb domain of TRF1 that is used by TRF1 to bind telomeric DNA. The Myb domain of TRF1 would therefore be inaccessible to U2AF65 when TRF1 is bound to DNA. Although U2AF65 interacts with a different domain of TRF1 than FBX4, which interacts with the TRFH domain of TRF1, TRF1 cannot interact with both proteins simultaneously (Kim and Chung, 2014). In addition, the shelterin component TIN2 also interferes with FBX4-mediated TRF1 turnover. TIN2 interacts with the TRFH dimerization domain of TRF1 (Ye and de Lange, 2004), preventing FBX4 association and thereby TRF1 ubiquitination and subsequent degradation (Zeng et al., 2010). TIN2 itself is also affected by ubiquitination. Its turnover is regulated by the E3 ligase SIAH2, which interacts with TIN2 to facilitate its proteasomal degradation (Bhanot and Smith, 2012). Finally, the F-box protein β-TRCP1 has also been shown to interact with TRF1 and promote its degradation. Similar to RLIM and FBX4, β-TRCP1 overexpression results in a reduced half-life of TRF1, while β-TRCP1 depletion leads to stabilization of TRF1 (Wang C. et al., 2013). Interestingly, β-TRCP1 overexpression also resulted in an increase in the percentage of APBs, which is surprising as TRF1 is known to be required for APB formation. Although an explanation could be that perhaps β-TRCP1 degrades not all but only a specific pool of TRF1, further studies are necessary to determine how TRF1 degradation by β-TRCP1 can be correlated with a function for β-TRCP1 in APB formation.

In contrast to the E3 ligases RLIM, FBX4 and β-TRCP1 promoting TRF1 turnover, the GCN5 and USP22 components of the chromatin modifying complex SAGA have been shown to oppose TRF1 ubiquitination (Atanassov et al., 2009). Depletion of either the histone acetyltransferase GCN5 or the DUB USP22 results in a decrease in TRF1 levels, which can be prevented by inhibition of the proteasome. GCN5 was found to be required for USP22 to properly associate with the SAGA complex and to be able to deubiquitinate TRF1 and prevent its turnover (Atanassov et al., 2009). In conclusion, the above-discussed studies indicate that TRF1 levels in cells are tightly regulated by numerous different proteins and on multiple levels.

### Ubiquitination- and SUMOylation-Mediated Regulation of TPP1, TRF2 and RAP1

Another shelterin subunit that is subjected to ubiquitin-mediated proteolysis is TPP1, which is evidenced by stabilization of TPP1 protein levels upon inhibition of the proteasome. Although the E3 ubiquitin ligases targeting TPP1 are still unknown, the DUB USP7 has been shown to interact with human TPP1 and to remove ubiquitin chains from its surface. While USP7 depletion did not affect proteasome-regulated TPP1 levels, USP7 might interact in a redundant manner with other DUBs to stabilize TPP1 (Zemp and Lingner, 2014). In mice, TPP1 ubiquitination by the E3 ligase RNF8 is also required for its stabilization at telomeres (Rai et al., 2011). However, in humans such a regulatory role of ubiquitination on TPP1, beyond regulation of its turnover, has not been observed. Changes in ubiquitination of TPP1 in humans have not resulted in aberrant TPP1 function nor have shown effects on TPP1 interaction with other proteins, such as TIN2, POT1 and telomerase (Zemp and Lingner, 2014). While this could be related to the use of overexpressed tagged TPP1 in human cells, it might also potentially indicate species differences in the extent of regulatory roles of ubiquitination on TPP1. Nevertheless, additional roles of human TPP1 ubiquitination may still await discovery.

Furthermore, the shelterin subunit TRF2 has also been shown to be ubiquitinated. TRF2 turnover is regulated by the E3 ligase SIAH1 as part of a positive feedback loop involving TRF2, ATM and p53 (Fujita et al., 2010). When telomere shortening causes loss of TRF2-mediated telomere protection, the ATM kinase is activated, which induces p53 activity and results in replicative senescence. Subsequently, p53 induces transcription of SIAH1, which targets TRF2 for proteasomal degradation. This results in increased p53 activation, further decreasing TRF2 levels through SIAH1-mediated ubiquitination of TRF2 (Fujita et al., 2010). In addition, a crosstalk between ubiquitination and SUMOylation has recently been observed to contribute to regulation of TRF2 (Her et al., 2015). The E3 SUMO ligase PIAS1 was identified as a novel TRF2-interacting protein and shown to SUMOylate TRF2. SUMOylated TRF2 is subsequently recognized by the SUMO-targeted ubiquitin ligase (STUbL) RNF4 through its SIM. This results in ubiquitination of TRF2 and subsequent proteasomal degradation (Her et al., 2015). This probably affects only a fraction of the total pool of TRF2 in the cell, as TRF2 is essential for chromosome end protection and extensive turnover of TRF2 would result in telomere uncapping.

Finally, in *Saccharomyces cerevisiae* (budding yeast) Rap1 has been shown to be SUMOylated and subsequently targeted for proteasomal degradation by the STUbL Uls1 (Lescasse et al., 2013). Loss of Uls1 was shown to result in accumulation of poly-SUMOylated Rap1 and telomere fusions. These fusions could be prevented by introduction of *rap1* alleles lacking SUMOylation sites. This indicates that accumulation of poly-SUMOylated Rap1 promotes telomere fusion and suggests that poly-SUMOylated Rap1 is non-functional in telomere protection. The proposed model suggests that Uls1 promotes ubiquitination and subsequent degradation of poly-SUMOylated Rap1, thereby allowing for recruitment of non-SUMOylated Rap1 that is able to protect chromosome ends from fusing through NHEJ (Lescasse et al., 2013). To what extent these results can be translated to mammalian systems remains unclear, as in budding yeast Rap1 interacts directly with telomeres and protects against NHEJ, while in mammals no direct interaction between RAP1 and telomeres is detectable but RAP1 is recruited by TRF2 and seems to mainly protect against HR.

Altogether, it has become clear that telomere maintenance does not only depend on the binding-capability of shelterin itself to the telomeric DNA, but also on its regulation by PTMs. Both features are important in facilitating protection of genome stability by telomeres. Although ubiquitination has been shown to contribute to telomere maintenance in multiple ways, emerging data show that SUMOylation also plays an important role in this process. Further studies are likely to provide additional insight in how these modifications affect and regulate telomere function. Additionally, it would be beneficial to verify the extent to which findings from yeast studies are conserved in mammalian systems.

## Telomere Elongation

The majority of cancer cells (± 90%), as well as many stem cells, express telomerase to elongate telomeres (Lazzerini-Denchi and Sfeir, 2016). Recently, it has been shown that in human embryonic stem cells (hESCs) telomere length is stabilized by a tight balance between telomere elongation through telomerase and telomere trimming by XRCC3 and NBS1 (Rivera et al., 2017). In tumors, telomere length is less stable, as telomere length between cancer cells is variable and telomerase extends most of the telomeres during every replication cycle (Martinez and Blasco, 2015). Although the majority of cancer cells maintains telomere length by activation of telomerase, a smaller number uses the ALT mechanism. During ALT, a HR-dependent mechanism copies telomeric DNA from a nearby template, resulting in telomere lengthening but also telomeres loss, which account for the heterogeneous telomere length typically observed in ALT cells (Pickett and Reddel, 2015).

Telomerase and ALT have been shown to be regulated by various PTMs, including ubiquitination and SUMOylation. Below we will discuss the roles of these two PTMs in regulation of telomerase activity and stability, and in ALT.

### Telomerase in the Spotlight

**FIGURE 3:**
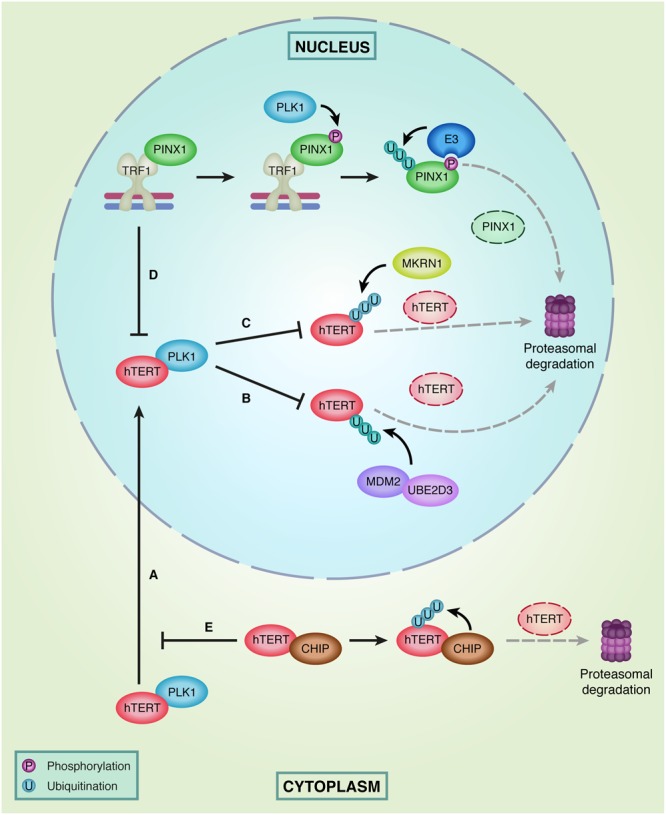
**Regulation of hTERT levels in the nucleus and cytoplasm. (A)** PLK1 facilitates hTERT localization to the nucleus. **(B)** In the nucleus PLK1 interferes with hTERT ubiquitination by MDM2 and UBE2D3 and subsequent degradation of hTERT by the proteasome. **(C)** In addition, PLK1 prevents hTERT proteasomal degradation by interfering with ubiquitination of hTERT by MKRN1. **(D)** hTERT binding to telomeres is prevented by TRF1, which is stabilized at telomeres by PINX1. However, PINX1 is a target of phosphorylation by PLK1, resulting in recruitment of E3 ligases that recognize phosphorylated PINX1. This promotes ubiquitination and proteasomal degradation of PINX1. **(E)** In the cytoplasm, the E3 ligase CHIP inhibits PLK1-facilitated transport of hTERT to the nucleus by interacting with hTERT. Subsequently, CHIP can ubiquitinate hTERT and target it for proteasomal degradation.

Especially the TERT subunit of telomerase has been shown to be modified by multiple ubiquitin E3 ligases, most of them regulating its proteasomal degradation (**Figure 3**). The first E3 ubiquitin ligase that was identified to interact with and ubiquitinate human (h) TERT is MKRN1 (Makorin-1 or RNF61) (Kim et al., 2005). Overexpression of this E3 ligase was shown to decrease telomerase activity and telomere length through ubiquitination and subsequent degradation of hTERT. In addition, MKRN1 has also been specifically implicated in modulation of telomerase activity during cell differentiation (Salvatico et al., 2010). The cancer cell line HL-60 normally expresses the *MKRN1* gene at very low levels and MKRN1 protein levels cannot be detected. However, upon retinoic acid induced differentiation of HL-60 cells MKRN1 expression significantly increased, coinciding with a strong down regulation of telomerase activity. As hTERT has a long half-life, MKRN1-mediated degradation of hTERT could provide efficient degradation of hTERT when telomerase activity is no longer needed (Salvatico et al., 2010).

A second E3 ubiquitin ligase that directly acts on hTERT is MDM2 (also known as HDM2). MDM2 and hTERT can physically interact through multiple domains on both proteins, whereby hTERT is polyubiquitinated and degraded by the proteasome. In line with this, depletion of MDM2 resulted in increased hTERT protein levels and increased telomerase activity (Oh et al., 2010). In addition, the E2 ubiquitin-conjugating enzyme UBE2D3 (or UBCH5C) has also been shown to regulate hTERT ubiquitination. Similar to MDM2 depletion, UBE2D3 depletion results in hTERT accumulation and increased telomerase activity (Wang W. et al., 2013; Gao et al., 2016; Yang et al., 2016). As it is known that UBE2D3 and MDM2 function together in the ubiquitination of p53 (Saville et al., 2004) and both were shown to regulate hTERT ubiquitination, it is tempting to speculate that this E2–E3 couple might also act together in the ubiquitination of hTERT.

A third E3 ligase that interacts with hTERT is the co-chaperone protein CHIP (C-terminus of Hsc70-interacting protein). In contrast to the ligases mentioned above, CHIP binds to the premature form of hTERT in the cytoplasm to inhibit its transport into the nucleus and subsequent integration into the telomerase complex. This inhibitory function of CHIP on the nuclear import of hTERT has been shown to occur independently from the ubiquitin ligase activity of CHIP, which requires its U-box domain. However, for ubiquitination and subsequent degradation of hTERT, the U-box and E3 ligase activity of CHIP are necessary (Lee et al., 2010). Furthermore, the interaction between CHIP and hTERT was found to peak in G2/M phase and decrease during S-phase, suggesting that degradation of hTERT by CHIP is cell cycle regulated and exerted when telomerase does not act on telomeres.

In addition to MKRN1, MDM2 and CHIP, Polo-like kinase 1 (PLK1) was recently identified to directly interact with hTERT, but with positive effects on hTERT levels (Huang et al., 2015). PLK1 overexpression was shown to prevent ubiquitination and proteasomal degradation of hTERT and to result in increased total protein levels of hTERT, including increased levels of nuclear hTERT and chromatin-bound hTERT. Therefore, it was suggested that PLK1 facilitates localization of hTERT to the nucleus and might interfere with the function or binding of E3 ligases, such as MKRN1 and MDM2, to hTERT. This then prevents hTERT’s proteasomal degradation and stabilizes telomerase activity (Huang et al., 2015). Interestingly, elevated PLK1 expression has been observed in several tumors. This might contribute to increased hTERT levels and excessive telomerase activity in cancer cells, thereby increasing the proliferative capacity of these cells (Holtrich et al., 1994).

Additionally, PLK1 has also been implicated in mediating the turnover of PINX1 (Wang et al., 2010). PINX1 is known as an hTERT inhibitor that directly interacts with TRF1 (Zhou and Lu, 2001). PINX1 binding to TRF1 has been shown to promote TRF1 association with telomeric DNA, thereby contributing to telomerase inhibition by decreasing accessibility of telomeric DNA to telomerase (Yoo et al., 2009). PLK1 can phosphorylate PINX1 and induce its ubiquitin-mediated proteasomal degradation. It has been proposed that phosphorylation of PINX1 by PLK1 stimulates the activity of E3 ubiquitin ligases, which subsequently ubiquitinate PINX1 and target it for degradation (Wang et al., 2010). When PINX1 is degraded, other factors can gain access to TRF1 and might affect the binding of TRF1 to telomeres, thereby increasing telomerase recruitment. One of these factors is the poly (ADP-ribose) polymerase Tankyrase 1 (TNKS1). TNKS1 has been shown to interact with TRF1 and to PARylate it, resulting in TRF1 dissociation from telomeres and subsequent degradation of TRF1 by the E3 ligase FBX4 (Smith et al., 1998; Ye and de Lange, 2004; Lee et al., 2006) (**Figure 2**). Consequently, telomerase gains access to telomeres, leading to telomere elongation. Interestingly, PLK1 can also phosphorylate TNKS1 directly, which stabilizes TNKS1 protein levels and thereby increases TRF1 degradation and telomerase activity (Ha et al., 2012). The shelterin protein TIN2 adds a final layer of complexity to this, as TIN2 can interact with TNKS1 and TRF1, thereby preventing TRF1 inactivation by TNKS1, resulting in TRF1 accumulation at telomeres and inhibition of telomerase loading (Ye and de Lange, 2004).

While ubiquitination evidently plays an important role in the regulation of telomerase activity in mammals, the contribution of SUMOylation to regulation of mammalian telomerase is not yet evident, despite the discovery of SUMOylation-dependent mechanisms of telomerase control in yeast. In *Schizosaccharomyces pombe* (fission yeast) SUMOylation of Tpz1, the homolog of TPP1, has been connected to negative regulation of telomere elongation (Hang et al., 2011; Garg et al., 2014; Miyagawa et al., 2014). SUMOylation of Tpz1 decreases telomerase binding to telomeres. This occurs through recruitment of the Stn1–Ten1 subunits of the CST complex, known to be involved in telomere length regulation and chromosome end capping by preventing telomerase accumulation at telomeres (Price et al., 2010). In contrast, Tpz1 has also been shown to interact with Ccq1-Est1 and thereby promote the recruitment and activity of telomerase, indicating a double role for Tpz1 in maintaining telomere length homeostasis (Miyoshi et al., 2008; Miyagawa et al., 2014). In budding yeast, the Stn1 protein has also been observed as a negative regulator of telomerase activity and telomere elongation. Stn1 interacts with the POT1 homolog Cdc13 and this interaction is increased when Cdc13 is SUMOylated, strengthening the inhibitory effect of Stn1 on telomerase. This increased interaction between Stn1 and Cdc13 has been associated with reduced telomerase levels, supporting a negative regulatory effect of SUMOylation on telomerase activity (Hang et al., 2011).

In conclusion, multiple different proteins and post-translational modifications, including ubiquitination, phosphorylation and PARylation, directly or indirectly affect the activity of telomerase. Whether there is also a role for SUMOylation in the control of telomerase regulation in mammalian cells still needs to be uncovered.

### Alternative Lengthening of Telomeres: Surviving without Telomerase

Ubiquitination and SUMOylation are also important for promoting telomere elongation by ALT in cells without telomerase activity. The shelterin protein TRF1 has been shown to be an important factor for ALT by promoting APB formation and localization of telomeres to PML bodies. To be able to facilitate this, TRF1 needs to be SUMOylated by MMS21, the E3 SUMO ligase component of the SMC5/6 complex (Potts and Yu, 2007). If MMS21-mediated SUMOylation is prevented, TRF1 recruitment to PML bodies is inhibited and APB formation is impaired. In addition to TRF1, also TRF2, TIN2 and RAP1 are SUMOylated by MMS21. When MMS21 is depleted, SUMOylation of all these shelterin subunits is inhibited (Potts and Yu, 2007). SUMOylation of multiple components of a complex could affect its stability; therefore, MMS21-mediated SUMOylation of shelterin components was suggested to induce disassembly of the shelterin complex within APBs. This could result in telomere deprotection, thereby potentially facilitating telomere recombination in ALT cells. As mentioned before, TRF1 can also be ubiquitinated by the E3 ligase β-TRCP1, promoting degradation of TRF1. However, β-TRCP1 has also been found to be essential for APB formation, as inhibition of β-TRCP1 decreased the percentage of APBs (Wang C. et al., 2013). This seems contradictory for a negative regulator of TRF1, but it has been suggested that β-TRCP1 only degrades telomere-unbound TRF1. If SUMOylation of the shelterin subunits indeed results in disassembly of the shelterin complex, β-TRCP1 might assist in the degradation of telomere-unbound TRF1.

Another factor involved in ALT is the scaffold protein SLX4, which recognizes DNA lesions and facilitates DNA repair by interacting with multiple endonucleases. Its structure includes both ubiquitin-binding zinc fingers (UBZs), involved in DNA interstrand crosslink repair, as well as SIMs that are important for localization of SLX4 to ALT telomeres (Ouyang et al., 2015). In addition, SLX4 contributes to telomere maintenance and protection by directly interacting with TRF2 via a motif resembling the TRF2-binding motif (TBM) present in other proteins known to interact with TRF2 (Wilson et al., 2013). The SUMO binding capacity of SLX4 was shown to enhance its interaction with several DNA damage sensors and telomere-binding proteins, including RPA, the MRE11-RAD50-NBS1 (MRN) complex and TRF2 (Ouyang et al., 2015). Therefore, increased SUMOylation of proteins at ALT telomeres may assist in the recruitment of SLX4 and other factors involved in ALT.

In budding yeast, telomere-bound proteins become increasingly SUMOylated when cells without telomerase activity obtain critically short telomeres that induce crisis. These SUMOylated proteins are recognized by the STUbL Slx5–Slx8, which mediates the relocalization of critically short telomeres to nuclear pore complexes (NPCs), where recombination events similar to the mammalian ALT pathway occur (Churikov et al., 2016). As Slx5–Slx8 can interact with SUMOylated telomere-bound proteins, as well as with the Nup84 complex of NPCs, it is believed to tether telomeres to the NPCs, thereby enabling recombination (Nagai et al., 2008; Churikov et al., 2016). Interestingly RNF4, the human homolog of Slx5–Slx8, has been shown to localize to PML bodies in human cells (Lallemand-Breitenbach et al., 2008; Tatham et al., 2008; Weisshaar et al., 2008). Although it is unknown whether RNF4 activity is necessary for ALT, it would be interesting to investigate if RNF4 has a similar function in humans as Slx5–Slx8 has in budding yeast. As discussed above, the MMS21 component of the SMC5/6 complex is necessary for SUMOylation at telomeres, which is needed for the recruitment of telomeres to PML bodies. RNF4 could potentially be involved in this by recognizing the MMS21-SUMOylated TRF1 at telomeres and promoting telomere recruitment to APBs.

Altogether, the above-discussed data show that ubiquitination and SUMOylation are crucial in the regulation of telomere elongation by telomerase and ALT. Multiple E3 ligases were shown to control the ubiquitination of hTERT to tightly regulate its levels and activity. In addition, various proteins and PTMs regulate the inhibitory function of TRF1 on telomerase. The complexity of telomerase regulation at telomeres suggests that this regulation is strictly controlled to prevent unscheduled access of telomerase to telomeres. However, further studies are necessary to understand how these mechanisms are coordinated and whether they are interrelated. In addition, ubiquitination and SUMOylation of shelterin components also seems to be crucial in promoting ALT, indicating that post-translational modification of shelterin components contributes to multiple processes involved in telomere maintenance and elongation.

## Telomere Deprotection

Successive rounds of cell division in the absence of telomerase or ALT ultimately lead to critically short telomeres and deprotected chromosome ends. These deprotected ends are recognized as damaged DNA by the DDR machinery. This results in activation of the p53 and Rb pathways and entry into senescence to limit telomere fusions and prevent subsequent genomic instability. However, when these pathways are impaired, senescence is bypassed and cells continue to divide and further lose telomere repeats until they reach crisis, a state of massive genome instability and cell death (d’Adda di Fagagna et al., 2003; Jacobs and de Lange, 2005; Palm and de Lange, 2008). At this stage, DNA repair pathways are activated and telomeres fuse in G1-phase either through Artemis and DNA ligase IV mediated c-NHEJ, or through alternative NHEJ (alt-NHEJ), a pathway mediated by PARP1 and DNA Ligase III. During subsequent cell divisions breakage-fusion-bridge cycles occur, inducing genomic instability and cell death. Nevertheless, a small portion of cells might escape crisis by reactivating telomerase or inducing ALT to maintain their telomeres, which results in expansion of cells with aberrant genomes, thereby promoting tumorigenesis (Arnoult and Karlseder, 2015; Lazzerini-Denchi and Sfeir, 2016).

### Signaling through the RNF8–RNF168 Pathway at Uncapped Telomeres

The DDR activated by deprotected telomeres in many ways resembles the DDR at general DNA double-strand breaks (DSBs). The DDR at telomeres starts with the recognition of uncapped telomeres by the MRN complex and activation of the ATM kinase, resulting in phosphorylation of histone H2AX at serine 139, generating γH2AX. This serves as a binding platform for MDC1, which initiates a positive feedback loop by promoting further accumulation of MRN and ATM (Peuscher and Jacobs, 2012). This results in spreading of γH2AX along the chromatin and amplification of DDR signaling and repair factor recruitment (Cimprich and Cortez, 2008; Brown and Jackson, 2015). At DSBs MDC1 is known to recruit RNF8, which interacts with phosphorylated MDC1 via its FHA domain (Kolas et al., 2007; Mailand et al., 2007). Depletion of either MDC1 or RNF8 causes a similar defect in the accumulation of 53BP1 at dysfunctional telomeres and reduces telomere fusions upon TRF2 inhibition (Dimitrova and de Lange, 2006; Peuscher and Jacobs, 2011). Furthermore, RNF8 requires its FHA domain to accumulate and promote NHEJ at uncapped telomeres, suggesting that RNF8 also recognizes phosphorylated MDC1 in this setting. Together this indicates that MDC1 and RNF8 function in the same pathway at telomeres and in a way that is identical to the DDR at genome-wide DSBs (Peuscher and Jacobs, 2011).

Upon RNF8 recruitment to uncapped telomeres, the RNF8/RNF168 signaling cascade is activated, promoting ubiquitination of histone H2A and subsequent recruitment of 53BP1. In addition, the recruitment of 53BP1 is dependent on the recognition of H4K20me2 by the Tudor domains of 53BP1 (Dimitrova et al., 2008). 53BP1 recruitment to telomeres results in accrual of RIF1 and MAD2L2 to promote NHEJ. This has been shown to block 5′ end-resection and HR through inhibition of BRCA1 recruitment to uncapped telomeres (Dimitrova et al., 2008; Chapman et al., 2013; Zimmermann et al., 2013; Boersma et al., 2015). The same mechanism has also been described at DNA DSBs and in immunoglobulin class-switch recombination (CSR) (Manis et al., 2004; Bothmer et al., 2010; Di Virgilio et al., 2013; Escribano-Diaz et al., 2013; Feng et al., 2013; Boersma et al., 2015; Xu et al., 2015). In addition, 53BP1 also recruits PTIP, which was also reported to contribute to fusion of dysfunctional telomeres. Furthermore, in BRCA1 deficient cells PTIP was shown to inhibit DSB resection and thereby promote genomic instability (Callen et al., 2013).

Depletion of RNF8 in cells with inactivated TRF2 leads to decreased H2A ubiquitination and 53BP1 recruitment at telomeres and a reduction in telomere fusions (Peuscher and Jacobs, 2011). In addition, inhibition of RNF168 recruitment to telomeres by the iDDR domain of TRF2 (part of the hinge domain) also results in a decrease in 53BP1 accumulation at dysfunctional telomeres (Okamoto et al., 2013). It has been shown that the iDDR domain of TRF2 prevents RNF168 recruitment to telomeres by accrual of the E3 ubiquitin ligase UBR5 and the MRN complex, resulting in inhibition of the signaling cascade downstream of ATM and protecting against telomere fusions through inhibition of NHEJ. UBR5 has been shown to function together with the E3 ligase TRIP12 at DSBs to control the levels and recruitment of RNF168 by targeting it for proteasomal degradation (Gudjonsson et al., 2012). The interaction of TRF2 with UBR5 could therefore inhibit RNF168 recruitment to telomeres. Interestingly, the MRN complex was shown to recruit the DUB BRCC3, which is part of the BRCA1-A complex and has been suggested to counteract the action of RNF8-UBC13 at DSBs (Shao et al., 2009). In this way, BRCC3 could inhibit recruitment of RNF168 to telomeres. Although BRCA1 is usually implicated in facilitating end-resection and HR, the BRCA1-A complex has also been suggested to restrict end-resection at DNA breaks (Coleman and Greenberg, 2011). In addition, BRCA1 has been shown to contribute to chromosome end protection (Al-Wahiby and Slijepcevic, 2005). Altogether, these studies indicate that also at dysfunctional telomeres the RNF8/RNF168 pathway promotes H2A ubiquitination and recruitment of 53BP1 to activate NHEJ.

Recently, RNF8 has been shown to ubiquitinate histone H1 at DSBs, together with the E2 ubiquitin-conjugating enzyme UBC13 (Thorslund et al., 2015). Ubiquitinated histone H1 serves as a binding platform for RNF168, which subsequently ubiquitinates histone H2A on K13/K15 and allows for recruitment of several repair factors, such as 53BP1 and BRCA1 (Mattiroli et al., 2012; Fradet-Turcotte et al., 2013; Thorslund et al., 2015). Furthermore, the DUB USP51 was shown to reverse the ubiquitination of histone H2A at K13/K15. Depletion of USP51 induces an increase in H2A K13/K15 ubiquitination and a delay in DDR foci resolution (Wang et al., 2016). However, many other DUBs have also been shown to be able to deubiquitinate histone H2A, including USP3, USP16, USP26 and USP44 (Citterio, 2015). Whether RNF8 ubiquitinates histone H1 at telomeres and whether RNF168 modifies histone H2A at K13/K15 at telomeres remains to be elucidated.

Finally, the STUbL RNF4 has also been shown to promote NHEJ at uncapped telomeres (Groocock et al., 2014). It usually recognizes SUMO-modified targets and is only activated upon dimerization in the presence of SUMO-chains (Rojas-Fernandez et al., 2014). RNF4 was suggested to promote 53BP1 recruitment to uncapped telomeres and telomere fusions, depending on a nucleosome-targeting motif in its RING domain and its SIMs (Groocock et al., 2014). This suggests that RNF4 recognizes chromatin-bound SUMO conjugates via its SIM domains (interaction with SUMO proteins) and its RING domain (binding to chromatin) and can subsequently ubiquitinate nearby chromatin or target proteins.

### Repression of the DDR and DNA Repair at Telomeres

In contrast to repair at genome-wide DSBs, which contributes to genome stability by fixing the break, repair at uncapped telomeres can be deleterious. When DNA repair factors gain access to chromosome ends and create end-to-end fusions in an attempt to ‘heal the break’, this can result in genomic instability. Therefore, telomeres need to be protected from unwanted actions of DNA repair factors. This protection is mainly achieved by the TRF2 and POT1 subunits of the shelterin complex (Doksani and de Lange, 2014). TRF2 has been shown to protect telomeres in at least five ways. First, TRF2 binding to telomeric DNA stimulates strand invasion and thereby T-loop formation, which hides telomere ends and prevents binding of the MRN complex and subsequent activation of ATM (Griffith et al., 1999; Stansel et al., 2001; Doksani et al., 2013). Secondly, TRF2 interferes directly with activation of the ATM kinase. TRF2 was found to be able to bind to ATM and prevent its phosphorylation at S1981, resulting in inhibition of the signaling cascade downstream of ATM (Karlseder et al., 2004). Thirdly, TRF2 has been shown to interact with the ATM target CHK2 at a position close to its Thr68 phosphorylation site, preventing activation of CHK2 (Buscemi et al., 2009). Fourthly, as discussed above, the iDDR domain of TRF2 prevents RNF168 and 53BP1 recruitment to telomeres, inhibiting the ATM signaling cascade downstream of ATM itself (Okamoto et al., 2013). Finally, TRF2 interacts with the α-helix 5 domain of Ku70, preventing Ku70–Ku80 heterotetramerization and activation of NHEJ (Ribes-Zamora et al., 2013). In addition to the protective function of TRF2, POT1 protects telomeres by repressing ATR activity. POT1 binds to the ssDNA of the 3′-overhang and is believed to prevent the recruitment of RPA, which is crucial for ATR activation (Gong and de Lange, 2010).

Although the shelterin complex protects telomeres throughout most of the cell cycle, telomeres are briefly deprotected during and after replication, before shelterin mediated protection has been re-established on newly replicated telomeres (Verdun and Karlseder, 2006). In addition, telomeres appear to be in an underprotected state during mitosis when the mitotic kinase Aurora B promotes telomere deprotection (Hayashi et al., 2012). This makes mitotic telomeres vulnerable to form sister-telomere associations (Orthwein et al., 2014). Therefore, during mitosis DNA repair is actively suppressed to prevent genomic instability. This suppression is achieved by CDK1-mediated phosphorylation of RNF8 and CDK1- and PLK1-mediated phosphorylation of 53BP1 (Orthwein et al., 2014). RNF8 phosphorylation interferes with the binding of RNF8 to MDC1, thereby inhibiting its recruitment to DSBs and preventing subsequent DDR signaling. In addition, phosphorylation of 53BP1 interferes with its ability to recognize H4K20me2 and K15-ubiquitinated histone H2A, which are both critical for 53BP1 recruitment to DSBs. This results in suppression of DNA repair activity during mitosis. When RNF168 and 53BP1 were artificially recruited to DSBs and telomeres, DNA repair was restored, resulting in sister telomere fusions (Orthwein et al., 2014). Thus phosphorylation of RNF168 and 53BP1 in mitosis plays an important role in maintaining genome stability and represents a shelterin-independent way of preventing DNA repair activities from acting on telomeres.

In general, it is increasingly becoming clear that ubiquitination and SUMOylation are highly involved in the regulation of the DDR triggered by DSBs in the genome. Evidence that this is also the case in response to dysfunctional telomeres has started to emerge, although there is still relatively little known about telomere-specific mechanisms. So far, the initial observations have indicated similar signaling processes in the cell’s response to DSBs and dysfunctional telomeres. However, also differences between these processes have been reported. For example at telomeres, in contrast to DSBs, the Ku70–Ku80 complex is not only recruited upon damage or uncapping, but is constitutively present to prevent deletion of telomeric repeats (Wang et al., 2009). However, it also promotes NHEJ at uncapped telomeres and is therefore restricted in its activity by TRF2. TRF2, as mentioned above, inhibits Ku70–Ku80 heterotetramerization, which interferes with Ku70–Ku80 activation and NHEJ induction, but does not deplete Ku from healthy telomeres (Ribes-Zamora et al., 2013). Furthermore, telomere deprotection has been shown to occur in different degrees, depending on the amount of TRF2 still bound to the telomeres (Cesare et al., 2013). Cells containing partially deprotected telomeres, with low amounts of TRF2 bound, will bypass the G2/M checkpoint, cycle to G1-phase and enter senescence, but will still be protected from telomeric NHEJ. However, when telomeres are completely uncapped, the DDR is fully activated and telomeres are fused through NHEJ (Cesare et al., 2013). Completely uncapped telomeres also avoid G2/M arrest, but the mechanism behind this is not yet known. These examples further emphasize that new findings regarding responses at DSBs, including ubiquitination and SUMOylation events, should also be studied at dysfunctional telomeres and vice versa, to understand whether the underlying mechanisms are identical or different between DSBs and dysfunctional telomeres.

## Perspectives

A tight regulation of telomere maintenance, replication and protection is required to ensure safeguarding of genome integrity by telomeres. If factors in these processes are impaired or exhibit aberrant functions, genome stability is at risk, potentially promoting tumorigenesis. Therefore, it is crucial to further investigate the processes and factors that ensure proper telomere function. Over the last decade, the importance and functions of ubiquitination and SUMOylation at telomeres have started to become clear. These PTMs are not only essential for telomere maintenance and protection, but are also key contributors to the cell’s response to dysfunctional telomeres. Although many studies have already explored ubiquitination and SUMOylation in different telomeric contexts and thereby identified various targets, the underlying mechanisms, as well as the precise contribution of PTMs are often still undetermined. Moreover, PTMs have also been shown to affect each other. So far, crosstalk between ubiquitination and SUMOylation has been shown to not only contribute to the general DDR, but also to DDR at telomeres, such as RNF4-mediated ubiquitination that was shown to require the presence of SUMO-chains (Groocock et al., 2014; Rojas-Fernandez et al., 2014). Therefore, it would be interesting to investigate whether additional crosstalk occurs at telomeres and if one aspect of telomere biology, for example DNA repair or maintenance, is more affected by the combination of ubiquitin and SUMO modifications than others. Further studies concerning telomere-specific ubiquitination and SUMOylation will be required to increase our understanding of the complex mechanisms that ensure proper telomere function or contribute to DNA repair at dysfunctional telomeres. It would be beneficial to distinguish which modifications are unique to telomeric DNA, as these might offer a tool to specifically target DNA repair at telomeres without interfering in an unwanted manner with genome-wide repair at DNA breaks.

## Author Contributions

All authors listed, have made substantial, direct and intellectual contribution to the work, and approved it for publication.

## Conflict of Interest Statement

The authors declare that the research was conducted in the absence of any commercial or financial relationships that could be construed as a potential conflict of interest.
